# ClpC2 protects mycobacteria against a natural antibiotic targeting ClpC1-dependent protein degradation

**DOI:** 10.1038/s42003-023-04658-9

**Published:** 2023-03-21

**Authors:** Gabrielle Taylor, Hengjun Cui, Julia Leodolter, Christoph Giese, Eilika Weber-Ban

**Affiliations:** 1 ETH Zurich, Institute of Molecular Biology & Biophysics, CH-8093 Zurich, Switzerland; 2 Present Address: Research Institute of Molecular Pathology (IMP), Vienna, Austria

**Keywords:** Bacterial transcription, X-ray crystallography, Protein quality control, Pathogens

## Abstract

*Mycobacterium tuberculosis* Clp proteases are targeted by several antitubercular compounds, including cyclomarin A (CymA). CymA exerts its toxicity by binding to AAA + chaperone ClpC1. Here, we show that CymA can also bind a partial homologue of ClpC1, known as ClpC2, and we reveal the molecular basis of these interactions by determining the structure of the *M. tuberculosis* ClpC2:CymA complex. Furthermore, we show deletion of *clpC2* in *Mycobacterium smegmatis* increases sensitivity to CymA. We find CymA exposure leads to a considerable upregulation of ClpC2 via a mechanism in which binding of CymA to ClpC2 prevents binding of ClpC2 to its own promoter, resulting in upregulation of its own transcription in response to CymA. Our study reveals that ClpC2 not only senses CymA, but that through this interaction it can act as a molecular sponge to counteract the toxic effects of CymA and possibly other toxins targeting essential protease component ClpC1 in mycobacteria.

## Abstract (web summary)

Mycobacteria evade the toxic effects of Cyclomarin A (CymA) by massive autoupregulated expression of the partial ClpC1 homologue ClpC2 that thereby acts as a tightly binding molecular sponge to prevent CymA binding to ClpCP.

## Introduction

*Mycobacterium tuberculosis* (Mtb), the aetiological agent of tuberculosis (TB), persists to this day as a major burden on global healthcare with multidrug-resistant and extensively drug-resistant strains compounding the difficulties in treating TB^1–3^. Consequently, it is necessary to resort to antibiotics that are more expensive and have lower efficacy with more side effects^4–6^. Therefore, novel antitubercular drugs together with a molecular understanding of their mechanism and cellular targets are needed to control the spread of TB.

Modular chaperone-protease complexes share a similar overall architecture consisting of a cylindrical proteolytic core that associates with a chaperone ring, thereby coordinating unfolding and proteolysis of specific protein substrates^7–10^. The caseinolytic protease (Clp) complexes are almost ubiquitous in bacteria, absent in only a few with minimal genomes^11,12^. In mycobacteria, the Clp chaperone-protease complexes feature a proteolytic core, ClpP, composed of both ClpP1 and ClpP2 subunits, that associates with an unfoldase, either ClpC1 or ClpX, to form the active ClpC1P or ClpXP proteases, respectively^13–15^. ClpC1 contains a mostly α-helical N-terminal domain (NTD) comprising the first 145 residues of ClpC1, followed by two AAA + (ATPases associated with various cellular activities) modules required for the energy-dependent substrate unfolding and translocation activity^16^. The NTD of ClpC1 and homologues associates with adaptor/regulatory proteins contributing to ClpC1P activity regulation and is critical for delivery of certain substrate classes^17–24^. In Mtb, ClpC1, ClpX and ClpP core subunits are all essential for cell viability, making the Clp system a promising drug target^25–27^.

Several compounds with antitubercular activity are known to target the mycobacterial Clp system^28^ and many of these compounds specifically target ClpC1 NTD, including lassomycin, ecumicin and cyclomarin A (CymA)^29–32^. CymA is a cyclic heptapeptide that is synthesised by the marine bacterium *Streptomyces* sp. CNB-982^33–35^. The compound is effective against mycobacteria but was found to show little activity against *Staphylococcus aureus* and *Escherichia coli*^34^. Biochemical and structural studies revealed CymA binds to the NTD of ClpC1 and it is this interaction that is responsible for CymA bactericidal activity^32^. In further studies, CymA was observed to enhance the ATPase and proteolytic activity as well as reduce substrate specificity of ClpC1 with subsequent deregulation of ClpC1P proteolytic activity leading to cell death^21,36^.

Mycobacteria also encode a ClpC1 partial homologue annotated as ClpC2. Given the sequence homology between ClpC1 NTD and ClpC2, we hypothesised CymA can also bind ClpC2. Through biochemical and structural studies, we show that ClpC2 presents a competitive binding site for CymA and is capable of sequestering CymA away from ClpC1. Furthermore, our in vivo investigations in *Mycobacterium smegmatis* show that the interaction between ClpC2 and CymA protects the organism against CymA-induced toxicity. In addition, we reveal that ClpC2 strongly upregulates its own transcription in response to CymA, shedding light on the regulatory mechanism by which mycobacteria counteract the toxic effects of ClpC1-targeting antibiotic compounds. Combined our results uncover and characterise a mycobacterial natural antibiotic resistance mechanism that senses and neutralises compounds targeting an essential protein degradation pathway.

## Results

### ClpC2 binds to the antitubercular compound CymA

**Fig. 1:**
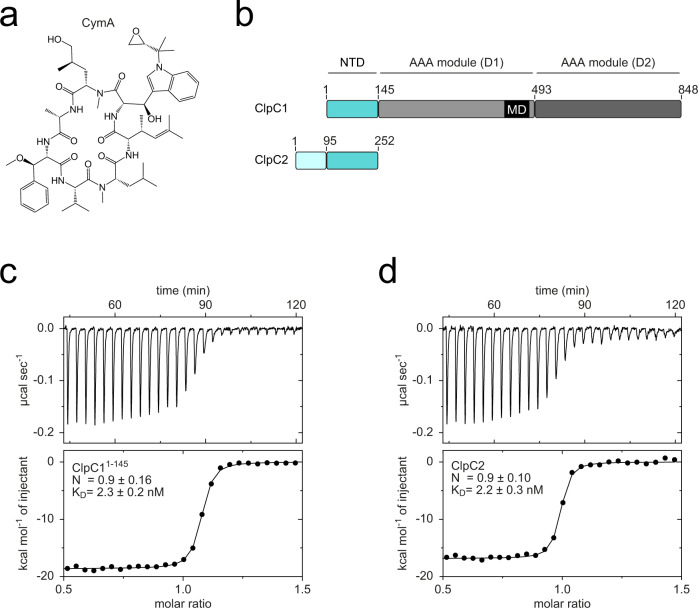
The antitubercular compound CymA binds to Mtb ClpC2. **a** Structural formula of cyclomarin A (CymA). **b** Domain organisation of Mtb ClpC1 alongside its partial homologue ClpC2. ClpC1 NTD, N-terminal domain (cyan); AAA, ATPases Associated with diverse cellular Activities (shades of grey); MD, middle domain (black). The ClpC1 partial homologue, ClpC2, is coloured according to sequence homology to ClpC1 NTD: homologous region (cyan); non-homologous region (light blue). **c**, **d** Injection profiles and binding isotherms for ITC titrations of ClpC1^1–145^ against CymA (**c**) and ClpC2 against CymA (**d**). The dissociation constant is indicated within each panel. Measurements were performed at 37 °C titrating aliquots of 50 μM ClpC1^1–145^ (**c**) or ClpC2 (**d**) to 5 μM CymA. *N* and *K*_D_ values are the mean ± standard deviation from three independent experiments.

The bactericidal peptide CymA (Fig. 1a) binds to the NTD of ClpC1^21,32,34,35^. In addition to ClpC1, many actinobacteria encode a ClpC1 partial homologue annotated as ClpC2 that shares sequence similarity with ClpC1 NTD (Fig. 1b). Given the resemblance between ClpC1 NTD and the ClpC2 C-terminal 160 residues (Supplementary Fig. 1), we hypothesised CymA can also interact with ClpC2. An alignment of the amino acid sequence of the Mtb ClpC1 NTD and the ClpC2 C-terminal region from several actinobacteria shows ClpC1 residues (F2, F80 and E89) critical for interaction with CymA are also conserved in ClpC2 (Supplementary Fig. 1)^32^.

To investigate the molecular interaction between ClpC2 and CymA, we heterologously expressed and purified Mtb ClpC2. The binding parameters between ClpC1 or ClpC1 NTD and CymA1, an amino-alcohol derivative of CymA, were previously analysed by isothermal titration calorimetry (ITC), producing dissociation constants (*K*_D_) values of 17.5 nM and 12 nM, respectively^32^. We used ITC to assess the affinity between CymA and ClpC2 and compared it to the affinity of the compound for ClpC1 NTD. For ClpC1 NTD and CymA, we observed a *K*_D_ in the low nanomolar range (2.3 nM) in ITC measurements (Fig. 1c), which is comparable to the value observed for the amino-alcohol derivative (CymA1). We determined a similar affinity for the binding of CymA to ClpC2 with a *K*_D_ of 2.2 nM and a stoichiometry of 1:1 (Fig. 1d). These data clearly indicate ClpC2, like ClpC1, binds tightly to CymA.

### ClpC2 dimerises via its N-terminal domain while the C-terminal domain binds CymA

**Fig. 2:**
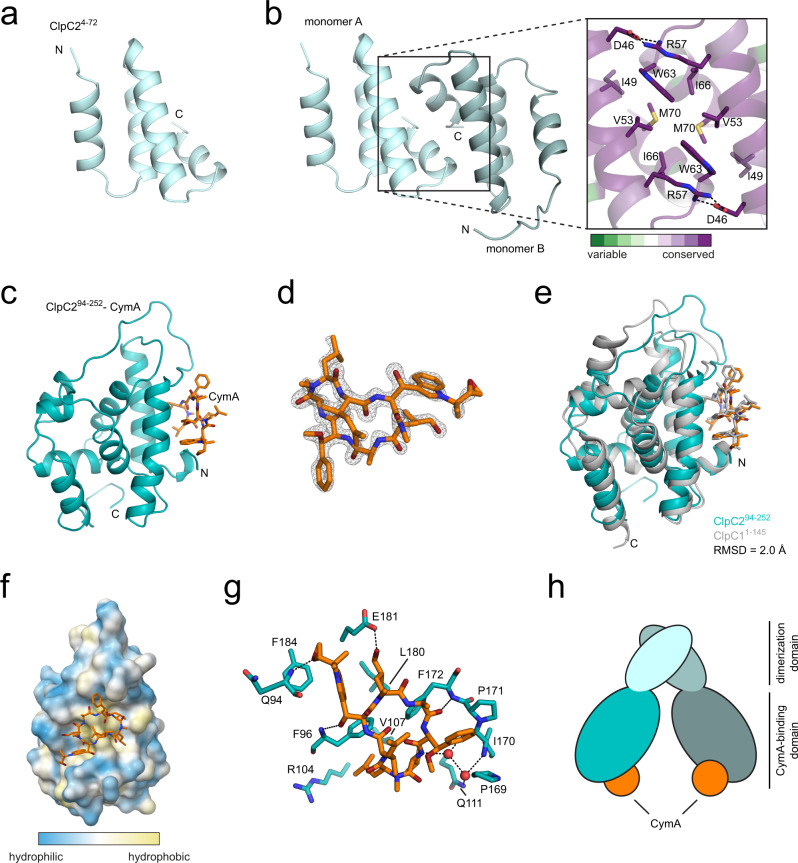
Crystal structure of the ClpC2 N-terminal domain and of the ClpC2 C-terminal domain in complex with CymA. **a** Crystal structure of the N-terminal domain of ClpC2 (ClpC2^4–72^). **b** ClpC2^4–72^ forms a homodimer in the crystallographic asymmetric unit (left panel) with each monomer coloured in light blue or light teal. In the right panel is a close-up view of the dimeric interface between ClpC2 monomers coloured according to the conservation of residues. Dashed lines suggest electrostatic interactions. **c** Overall structure of ClpC2 C-terminal domain (ClpC2^94–252^; cyan) bound to CymA (orange). **d** The unbiased mF_o_-DF_c_ Fourier map of CymA at 3.0 σ was calculated following molecular replacement using PDB 3WDC as a search model excluding ligands. **e** ClpC2^94–252^ (cyan) bound to CymA (orange) is superimposed onto ClpC1 NTD bound to CymA (PDB 3WDC; grey) showing a root mean square deviation (RMSD) of 2.0 Å. **f** CymA (orange, stick representation) bound to ClpC2^94–252^ (surface representation), coloured according to hydrophobicity. **g** Detailed molecular interactions between CymA and ClpC2 C-terminal domain. Shown in stick representation are the residues of ClpC2 (cyan) that are in close proximity to CymA (orange) with suggested hydrogen bond interactions represented by dashed lines and water molecules represented as red spheres. **h** A cartoon model of ClpC2 dimer colour-coded according to the presented ClpC2 crystal structures.

To obtain a molecular understanding of ClpC2 and its interaction with CymA, we set up crystallisation experiments with ClpC2 alone and in the presence of CymA. We determined the structure de novo using the programme ARCIMBOLDO^37^ for ab initio phasing, since molecular replacement failed, and refined it to ~2.0 Å resolution. Due to proteolysis in the crystallisation drop, the determined structure included ClpC2 residues 4–72 (ClpC2^4–72^), the region of ClpC2 without homology to ClpC1, but not the C-terminal portion of ClpC2 (Table 1, Fig. 2a). ClpC2^4–72^ forms a dimer in the asymmetric unit, with each monomer featuring a four-helix bundle (Fig. 2b). Analysis of the structure using PDBePISA^38^ strongly suggests dimerisation between the two N-terminal fragments with a total buried surface area of 1068 Å^2^. The intermolecular interactions within the dimerisation interface are primarily mediated by highly conserved hydrophobic residues and two salt bridges established by the D46-R57 pair (Fig. 2b). To confirm that the interface observed in the crystal structure reflects a homodimer interaction also occurring in solution, a ClpC2 R57A variant was generated and both ClpC2 WT and ClpC2 R57A were analysed by size-exclusion chromatography. Compared to the elution positions of size standard proteins, ClpC2 WT (monomeric molecular weight 26 kDa) appears to exist in an equilibrium between monomer and dimer at this concentration whereas the ClpC2 R57A variant elution profile corresponds to the monomeric species (Supplementary Fig. 2).

**Table 1:** Data collection and refinement statistics.

	ClpC2^4–72^	ClpC2^94–252^-CymA
PDB ID	8ADA	8AD9
Crystal form		
Space group	*P 3*_*1*_ *1 2*	*C 1 2 1*
Unit cell	48.14	142.582
Dimensions (Å)	48.14	40.455
	110.018	59.741
Angles α, β, γ (°)	90 90 120	90 112.467 90
Molecules/ASU	2	2
Data collection		
Wavelength (Å)	1.000	1.000
Resolution (Å)	38.99–1.996	34.72–1.43
	(2.067–1.996)	(1.481–1.43)
Total reflections	97 566 (8 690)	393 194 (36 160)
Unique reflections	10 152 (944)	57 671 (5 598)
Multiplicity	9.6 (9.2)	6.8 (6.5)
Completeness (%)	99.56 (96.33)	98.46 (96.22)
Mean *I*/*σ*(I)	14.82 (2.02)	14.54 (1.94)
Wilson B-factor (Å^2^)	32.77	21.96
*R*_merge_	0.089 (1.128)	0.056 (0.885)
*R*_meas_	0.095 (1.194)	0.061 (0.962)
CC_1/2_	0.998 (0.734)	0.998 (0.838)
Refinement		
Reflections used	10 151 (944)	57 671 (5 593)
Reflections used *R*_free_	525 (49)	2 842 (283)
*R*_work_	0.1798 (0.2703)	0.1801 (0.2834)
*R*_free_	0.2095 (0.2821)	0.2109 (0.2899)
Model composition		
Non-hydrogen atoms	1 068	2 695
Macromolecules	1 016	2 415
Ligands	0	59
Water	52	221
Protein residues	132	301
RMSD		
Bonds	0.010	0.013
Angles	1.03	1.14
Ramachandran plot		
Favoured (%)	99.22	99.66
Allowed (%)	0.78	0.34
Outliers (%)	0.00	0.00
Rotamer outliers (%)	0.93	0.00
Clashscore	2.47	2.69
Average B-factor	38.85	28.98
Macromolecules	38.56	28.03
Ligands	N/A	36.39
Water	44.62	37.38

Values in parentheses are for the highest-resolution shell.

${{{\mbox{R}}}}_{{{\mbox{merge}}}}=\sum \left|{I}_{h,i}-\left\langle {I}_{h}\right\rangle \,\right|/\sum {I}_{\left(h,i\right)}$, where $\left\langle {I}_{h}\right\rangle$ is the mean intensity of the reflections.

To also obtain structural information on the ClpC2 C-terminal, CymA-binding domain, we then constructed a ClpC2 truncation variant covering ClpC2 residues 94–252 that are homologous to ClpC1 NTD based on the sequence alignment (see Supplementary Fig. 1). This ClpC2 truncation variant (ClpC2^94–252^) was successfully co-crystallised with CymA and the structure of the complex was determined by molecular replacement using the ClpC1 NTD structure (PDB 3WDC) as a search model. The final atomic model of ClpC2^94–252^ bound to CymA was refined to a resolution of 1.43 Å (Table 1, Fig. 2c, d). Like the ClpC1 NTD, ClpC2^94–252^ forms two helical repeats and it binds CymA in an analogous fashion (Fig. 2e)^32^. The binding of CymA to ClpC2^94–252^ mainly stems from hydrophobic interactions that are largely mediated by the two highly conserved phenylalanine residues (F96 and F172; Fig. 2f, g). The two residues also contribute polar contacts through their backbone amides to the carbonyls of the N-reverse prenylated tryptophan and alanine of CymA with additional polar contacts provided by E181 and Q94 (Fig. 2g). Taken together, the structures we have solved in this study suggest ClpC2 dimerises via its N-terminal domain with the C-terminal domain harbouring the CymA-binding site (Fig. 2h).

### ClpC2 protects against CymA-induced toxicity

**Fig. 3:**
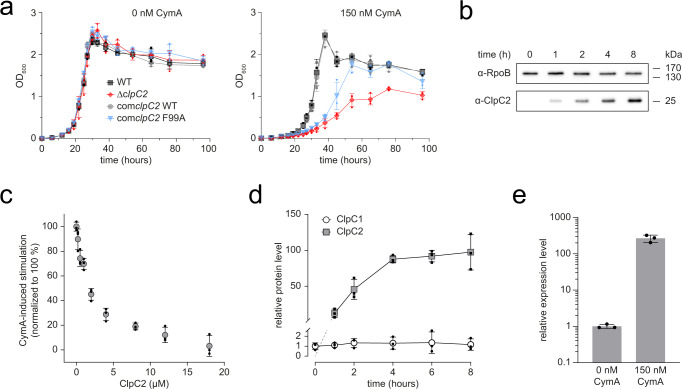
ClpC2 protects Msm against CymA toxicity. **a** A *clpC2* knockout (Δ*clpC2*) strain was prepared in Msm. The Δ*clpC2* (red diamonds), parent (WT, black squares) as well as Δ*clpC2* strains complemented with *clpC2* WT (com*clpC2* WT, grey circles) or complemented with *clpC2* F99A (com*clpC2* F99A, blue triangles) were grown to exponential phase, then diluted to an OD_600_ of 0.005 before monitoring the OD_600_ of the cultures grown in either the absence or presence of 150 nM CymA. Error bars depict the standard deviation from three biological replicates. **b** The effect of CymA on cellular ClpC2 protein levels was analysed by Western blot. Msm culture was grown to an OD_600_ of 0.5 in 7H9 medium at 37 °C before adding 150 nM CymA. Cells were harvested, then lysed at the indicated time points following addition of CymA. RpoB is included as a loading control. **c** The counteractive effect of ClpC2 in a CymA-stimulated FITC-casein degradation assay. The proteolytic activity of 0.5 µM ClpC1 hexamer/0.8 µM ClpP1P2 tetradecamer was stimulated by 2 µM CymA and the rate of degradation was determined following the addition of increasing concentrations of ClpC2. The rate of degradation is normalised so that 0% stimulation is the proteolytic activity in the absence of CymA and 100% stimulation is equivalent to the maximum CymA-induced stimulation above basal activity. Error bars represent standard deviation from three independent experiments. **d** Quantitative Western blot was employed to analyse the ratio of ClpC1 (open circles) to ClpC2 (filled squares) following addition of CymA. Msm culture was grown to an OD_600_ of 0.5 in 7H9 medium at 37 °C before adding 150 nM CymA. Samples were harvested at the indicated time points following addition of CymA and ClpC1 or ClpC2 proteins quantified by immunoblotting in reference to a protein standard curve using purified ClpC1 and ClpC2 protein. ClpC1 and ClpC2 protein levels are normalised relative to ClpC1 at timepoint 0 h. Representative blots are shown in Supplementary Fig. 3. Error bars depict standard deviation from biological triplicates. **e** Expression levels of *clpC2* in the absence and presence of 150 nM CymA as analysed by RT-qPCR. Msm cultures were grown to an OD_600_ of 0.5 followed by a one-hour incubation in either the absence or presence of 150 nM CymA. *clpC2* gene expression was normalised to the housekeeping gene *rpoB*. Error bars represent standard deviation of technical triplicates from three biological replicates. Statistical analysis by two-tailed unpaired Student’s *t* test; *P* value = 0.0015.

Like Mtb, *Mycobacterium smegmatis* (Msm), which is used as a model system for studying Mtb biology, is also sensitive to CymA and it encodes a ClpC2 homologue. Therefore, we turned to Msm to analyse the contribution of ClpC2 to cell viability in the absence and presence of CymA. We generated a *clpC2* knockout strain (Δ*clpC2*) in Msm which displayed growth behaviour similar to the parent strain (WT) under standard growth conditions (Fig. 3a). However, upon addition of sub-MIC_50_ concentrations of CymA^32^, Δ*clpC2* exhibited diminished growth compared to the parent strain, and the WT phenotype was restored in a *clpC2* complementary strain (com*clpC2 WT*) (Fig. 3a). To assess whether these effects originate from CymA-binding to ClpC2, we complemented the Δ*clpC2* strain with a CymA-binding-impaired variant. Previously, F2 was identified as a key residue for CymA-binding to Mtb ClpC1^32^, and our structural studies show the equivalent residue in Mtb ClpC2 (F96) contributing to CymA-binding (Fig. 2g). Therefore, we complemented Δ*clpC2* with *clpC2* F99A (the Msm equivalent to F96), and we observed attenuated growth upon exposure to CymA (Fig. 3a). The observed phenotype suggests ClpC2 protects mycobacteria from CymA-induced toxicity by directly binding the toxic compound.

### ClpC2 is upregulated in response to CymA

To understand further the ClpC2 response to CymA, we explored the effect of CymA on intracellular ClpC2 protein levels in WT Msm. We added sub-MIC_50_ concentrations of CymA to mid-log phase Msm cultures and followed the protein levels by Western blot. While the RpoB loading control remains constant throughout, no band for ClpC2 is detected in the Western blot in the absence of CymA, and the signal increases by several orders of magnitude from a faint band at 1 hour post CymA addition to 8 h following addition of CymA (Fig. 3b).

CymA was shown to have a stimulatory effect on ClpC1-dependent degradation of the model substrate casein^21,36^. Given the strong upregulation of ClpC2 in vivo, we decided to investigate the impact of ClpC2 on a CymA-stimulated degradation assay using fluorescein isothiocyanate-conjugated casein (FITC-casein). Adding increasing concentrations of ClpC2 counteracted the stimulatory effect of CymA on casein degradation (Fig. 3c), suggesting ClpC2 can sequester CymA, preventing it from binding ClpC1 and thus from modulating ClpC1 activity. In order to protect ClpC1 by competing for CymA, ClpC2 would have to reach cellular concentrations in excess of ClpC1. To compare the in vivo protein levels of ClpC1 and ClpC2, we quantified ClpC1 and ClpC2 protein levels at various time points following addition of CymA by quantitative Western blot. ClpC1 levels remained fairly constant throughout the 8-hour timeframe. In contrast, ClpC2 levels changed considerably starting at undetectable levels immediately after addition of CymA and rising to 84-fold in excess of ClpC1 around 8 h after addition of CymA (Fig. 3d, Supplementary Fig. 3). This suggests Msm can produce substantial quantities of ClpC2 well in excess of ClpC1 to sequester the toxic CymA.

Considering the large upregulation in ClpC2 protein, we next analysed the effect of CymA on ClpC2 transcription. Therefore, we grew Msm cultures in either the absence or presence of CymA and extracted total RNA for analysis of *clpC2* transcript levels by RT-qPCR. Comparison of relative transcript levels reveals a 263-fold increase in *clpC2* mRNA following exposure to sub-MIC_50_ concentrations of CymA for one-hour (Fig. 3e). Given the dramatic increase in *clpC2* expression, we directed our attentions to the influence of CymA on *clpC2* transcription.

### ClpC2 acts as a transcription factor to repress its own expression

We employed a DNA pull-down assay based on the protocol reported in Chaparian and van Kessel^39^ to identify the transcription factor responsible for the upregulation of ClpC2 in response to CymA (Supplementary Fig. 4a). The DNA probe used in the pull-down assay encompassed 300 bp of the DNA sequence upstream of the Msm *clpC2* start codon to ensure it included the putative promoter region. As a control, a stretch of intragenic Msm DNA sequence of similar length was used. Msm lysate was prepared from cultures grown in either the presence or absence of CymA and served as the source for the unknown transcription factor. Proteins retained with the DNA through affinity purification were subjected to tryptic digestion and analysed by liquid chromatography followed by tandem mass spectrometry (LC-MS/MS). Approximately 800 proteins were detected (Supplementary Data 1) many of which are suspected false positives. One of the proteins detected in the DNA pull-down assay was ClpC2 itself. Surprisingly, ClpC2 also was detected in the pull-down carried out in the absence of CymA (Supplementary Fig. 4b), where it was even slightly enriched. This was very unexpected considering the comparatively low ClpC2 protein levels we measured in absence of CymA (Fig. 3b, d), and suggested a potential role for ClpC2 in regulating its own expression.

**Fig. 4:**
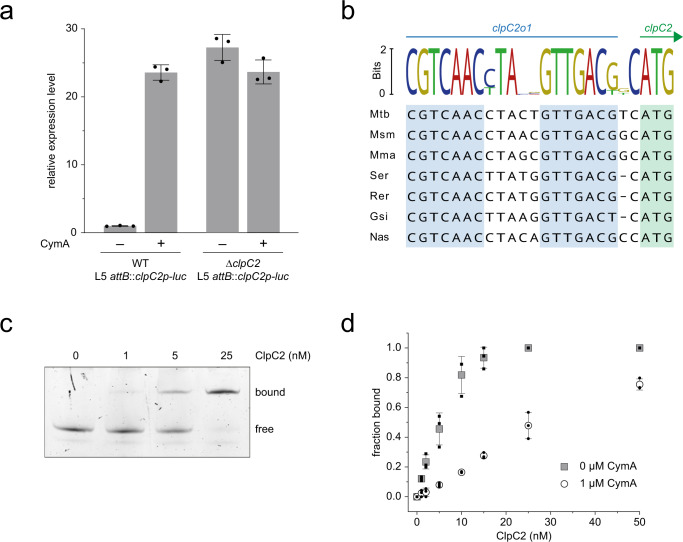
ClpC2 acts as a transcription factor regulating its own expression. **a** The luciferase gene under the control of the *clpC2* promoter was introduced into the genomes of both WT and Δ*clpC2* strains via an integrative vector (see Supplementary Fig 5). Cells were harvested following the addition of CymA and the total RNA was extracted in preparation for luciferase transcript levels analysis by RT-qPCR. Luciferase transcript levels are normalised to the housekeeping gene *rpoB* and expression levels are expressed relative to the wild-type strain grown in the absence of CymA. Error bars depict standard deviation from three biological replicates with statistical significance determined by two-tailed unpaired Student’s *t* test. **b** Alignment of DNA sequences immediately upstream of the *clpC2* open reading frame in a selection of actinobacteria. The start codon for *clpC2* and homologues is shaded in light green. Pseudopalindromic sequence regions upstream of the start codon are shaded in light blue. The sequence logo is displayed above the alignment. Mtb, *Mycobacterium tuberculosis*; Msm, *Mycobacterium smegmatis*; Mma, *Modestobacter marinus*; Ser, *Saccharopolyspora erythraea*; Rer, *Rhodococcus erythropolis*; Gsi, *Gordonia sihwensis*; Nas, *Nocardia asteroids*. **c** ClpC2 DNA-binding activity was analysed by electrophoretic mobility shift assay (EMSA). 1–25 nM ClpC2 was added to 0.5 nM DNA that includes the putative *clpC2* operator sequence. Data are representative of three independent experiments. **d** The binding of DNA to ClpC2 was analysed in the absence (filled squares) and presence (open circles) of 1 µM CymA. Gel band intensity was quantified by densitometry to determine the fraction of DNA bound to ClpC2. Representative gels are shown in Supplementary Fig. 8. Error bars represent the standard deviation of three independent experiments.

To investigate how ClpC2 might autoregulate its expression, both WT and Δ*clpC2* strains were transformed with an integrative plasmid that encodes the luciferase (*luc*) gene under the control of the *clpC2* promoter (see Supplementary Fig. 5). The effect of CymA on *luc* transcript levels was then followed by RT-qPCR for both strains. In agreement with Fig. 3e, luciferase expression in the modified WT culture was significantly higher in the presence of CymA compared to in the absence of CymA (Fig. 4a). This pattern of luciferase expression was not observed for the modified Δ*clpC2* cultures (Fig. 4a). Here, luciferase expression levels in the absence of CymA are already at the high level of expression that the modified WT cultures only reached in the presence of CymA. These results suggest that ClpC2 is required for repression of its own expression under standard growth conditions and that this transcriptional repression is lifted following the addition of CymA. The most direct role ClpC2 could play to achieve this effect is by acting as a transcriptional repressor of its own promoter.

We analysed the DNA sequence upstream of the *clpC2* open reading frame (ORF) in several actinobacterial organisms to determine the *clpC2* operator DNA sequence and identified a conserved pseudopalindromic sequence one to two nucleotides upstream of the *clpC2* ORF, which takes the form of CGTCAAC-n_5_-GTTGACG; where n is any nucleotide (Fig. 4b). The same palindromic sequence is repeated further upstream of the *clpC2* ORF in many of the actinobacterial organisms analysed, and together these operator sites overlap with the putative −35 and −10 elements of the *clpC2* promoter (Supplementary Fig. 6a). Additionally, the positioning of the −35 and −10 elements with respect to the *clpC2* ORF suggests *clpC2* transcripts are leaderless.

### CymA lifts transcriptional repression

The putative *clpC2* operator DNA sequence was incorporated into a DNA probe used in an electrophoretic mobility shift assay (EMSA). Titrating ClpC2 to the DNA probe produces a gel band shift confirming ClpC2 can bind the DNA (Fig. 4c). In the actinobacterial genomes containing two consecutive *clpC2* operator sites (e.g., Msm), ClpC2 can bind both sites simultaneously presumably leading to more stringent regulation of *clpC2* expression (Supplementary Fig. 6b). Furthermore, specificity for the *clpC2* operator sequence was demonstrated with the addition of either nonspecific or specific competitor DNA to labelled *clpC2* operator DNA (Supplementary Fig. 7a and 7b). The palindromic nature of the operator sites suggests the high-affinity interaction with DNA observed in Fig. 4c involves the ClpC2 dimer. Analysis of DNA binding by the dimerisation-deficient ClpC2 R57A and ClpC2 CTD variants in EMSA experiments provided further support for the importance of ClpC2 dimer formation for the high-affinity interaction with the *clpC2* operator site (Supplementary Fig. 7c and 7d). To explore the effects of CymA on DNA-binding, we performed the EMSA across a broader ClpC2 concentration range in the absence and the presence of CymA (Fig. 4d, Supplementary Fig. 8). CymA had a noticeable inhibitory effect on the interaction between ClpC2 and its operator site, providing the explanation for the lifting of transcriptional repression observed in Figs. 3e and 4a.

**Fig. 5:**
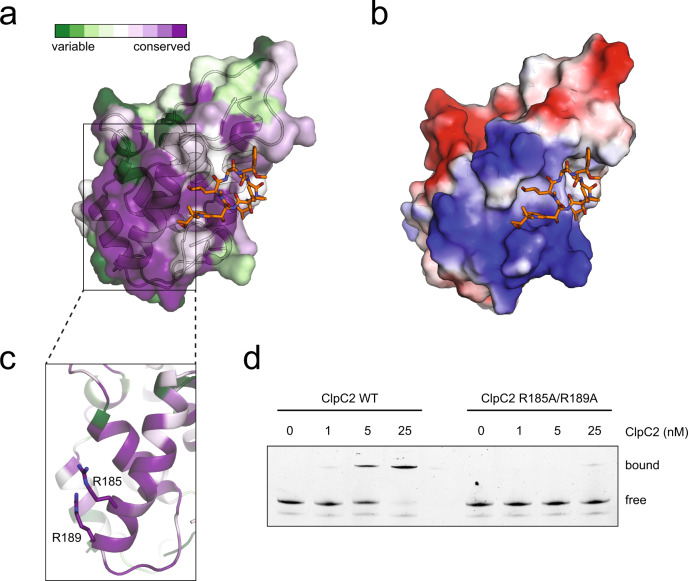
CymA interferes with Mtb ClpC2 binding to its promoter DNA. **a** Surface representation of ClpC2^94–252^-CymA coloured according to residue conservation produced using ConSurf^60–62^. CymA is coloured in orange and shown in stick representation. **b** Structure of ClpC2^94–252^ depicting surface charge and shown in the same orientation as in **a**. **c** Close-up of the suggested DNA recognition helix shown in the forefront. Arginines suggested to contribute to DNA-binding are shown in stick representation. **d** Comparison of ClpC2 WT and ClpC2 R185A/R189A double mutant in an electrophoretic mobility shift assay. ClpC2 variants were titrated against 0.5 nM DNA probe and loaded onto a 12% polyacrylamide gel. Data are representative of three independent experiments.

In order to rationalise such a mechanism of CymA-dependent derepression on the molecular level, we turned again to the structure of ClpC2^94–252^-CymA to identify the putative site for DNA binding. We observed a highly conserved surface region in the ClpC2 C-terminal domain (Fig. 5a) that coincides with a positively charged region (Fig. 5b). Contributing to the high degree of conservation and charge is a prominent helix (residues 174-189) that features two conserved arginines (R185 and R189; Fig. 5c). Therefore, to explore the potential for this region of ClpC2 to bind DNA, we tested a ClpC2 R185A/R189A variant in an EMSA experiment and observed the double mutant is unable to bind DNA as efficiently as ClpC2 WT (Fig. 5d). As R185 and R189 are conserved in Msm ClpC2 (R188 and R192), we analysed the *clpC2* mRNA levels of Δ*clpC2* complemented with either *clpC2* R188A/R192A (com*clpC2* R188A/R192A) or *clpC2* R57A (com*clpC2* R57A), the mutant that exhibited impaired dimerisation behaviour (Supplementary Fig. 2). Both, complementation with *clpC2* R188A/R192A and with *clpC2* R57A showed increased expression levels of 2.0-fold and 4.6-fold, respectively, compared to complementation with ClpC2 WT (Supplementary Fig. 9a). The knockout strain complemented with *clpC2* R188A/R192A showed similar growth behaviour as the knockout strain complemented with *clpC2* WT, indicating that the defect in repression of *clpC2* does not interfere with the protective effect of ClpC2 against CymA toxicity and also does not lead to a growth defect in rich medium under standard culture conditions (Supplementary Fig. 9b). Interestingly, although the arginine double-mutant can clearly still bind CymA, as evidenced by the lack of growth impairment observed with this mutant in the presence of CymA, the putative DNA-binding site is quite close to and might overlap partially with the CymA-binding site (Fig. 5a, b). Therefore, we hypothesise CymA either prevents DNA binding directly by sterically occluding the DNA-binding site, or CymA induces an alternative configuration of ClpC2 C-terminal domains that is incapable of interacting efficiently with DNA.

### Expression of *clpC2* is also induced under heat stress

In order to investigate if other stressors might alleviate *clpC2* repression, we tested *clpC2* mRNA levels under heat shock conditions. Inducing heat shock by increasing the temperature to 45 °C caused an increase in *clpC2* expression of ~twofold (Supplementary Fig. 10a). This suggests that stresses affecting ClpC2 stability might also lead to derepression and hence upregulation of ClpC2. ClpC1 is known to degrade unstructured model substrates like casein that can serve as a mimic for heat-destabilised proteins. Given the homology between the ClpC2 CTD and ClpC1 NTD, it is possible that ClpC2 might also bind disordered proteins. We, therefore, tested the effect of an excess of ClpC2 on ClpC1-dependent casein degradation. In the presence of ClpC2, FITC-casein degradation is slowed with a proportion of the substrate population protected from degradation, implying that at high micromolar concentrations, ClpC2 can bind FITC-casein and prevent degradation of the substrate by the ClpC1P complex (Supplementary Fig. 10b). These results demonstrate that ClpC2 shares some protein binding partners with ClpC1.

## Discussion

**Fig. 6:**
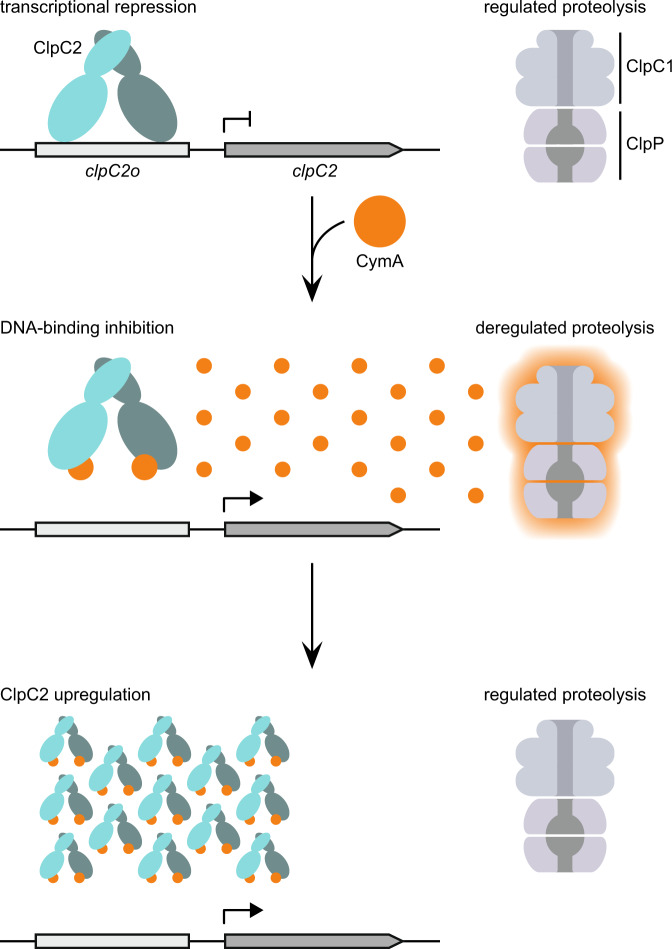
ClpC2 response to CymA exposure helps protect mycobacteria from CymA-induced toxicity. ClpC2 binds to its own operator site (*clpC2o*) performing the role of a transcriptional repressor under standard growth conditions. Upon exposure to CymA, both ClpC1 and ClpC2 bind CymA leading to both aberrant ClpC1P activity as well as the lifting of *clpC2* gene repression resulting in the upregulation of ClpC2. The increased ClpC2 protein levels bind the available CymA, preventing the toxic consequences of CymA interaction with ClpC1.

Mtb Clp proteases are considered to be promising drug targets with both inhibition and activation being deleterious to the organism. As such, these proteases have already been implicated in the mechanism of several natural products exhibiting mycobactericidal activity^28,29,40^. However, relatively little is known about the defensive response of the bacteria to the relevant antibacterial compounds. Here we report a role for the ClpC1 partial homologue ClpC2 in mediating against CymA-induced toxicity in mycobacteria. Furthermore, we can ascribe a mechanism by which ClpC2 binds DNA and represses its own expression under standard growth conditions. When presented with CymA, ClpC2 binds to CymA, hindering the interaction of ClpC2 with its own operator site and leading to loss of repression and subsequent upregulation of ClpC2. This upregulation of ClpC2 increases the capacity to bind free CymA, reducing the CymA available to interact with ClpC1 and therefore alleviating CymA-induced toxicity (Fig. 6).

Our ITC titrations and ClpC1P in vitro degradation assay suggest ClpC2 can bind CymA as well as compete with ClpC1 for the binding of CymA (Figs. 1d and 3c). However, CymA is only one of several antitubercular peptides that are known to bind to ClpC1 NTD. Co-crystal structures of ClpC1 NTD with ecumicin and rufomycin revealed overlapping binding sites with CymA, despite opposing effects on ClpC1P proteolytic activity^30,31,41,42^. Based on our findings, it appears plausible that other cyclic peptides may also bind to ClpC2. These compounds are generally of natural origin and are likely encountered by ClpC2-expressing actinobacteria in their natural habitats. Therefore, one possible explanation that fits with the data we present is that ClpC2 has the role to protect ClpC1 from these compounds by providing a competitor pool of binding sites for the toxins, titrating them away from interaction with ClpC1. Alternative sources for toxic ClpC1 interactors might be bacteriophages, as phage-encoded proteins were previously reported to deregulate protein degradation by binding to the NTD of the ClpC1 homologue in *Bacillus subtilis*^43^. Furthermore, ClpC2 may help to regulate the delivery of endogenous proteins to the Clp complexes by competing for specific substrates or substrate adaptors that usually dock at the ClpC1 NTD. For example, by inhibiting the degradation of specific substrates, ClpC2 might prevent adverse downstream cellular effects upon exposure to certain stressors. Our results with the model substrate casein support this notion and suggest a role for ClpC2 under heat stress, where it might act as a “holdase” chaperone to alleviate ClpC1 overload.

The upregulation of ClpC2 was previously observed in several transcriptional profiling studies into regulation during nutrient starvation, lysosomal stress and in persister/latent cells in Mtb^44–49^. In our study, we observed significant upregulation of ClpC2 in response to CymA and to a milder degree also under heat shock. With the finding that ClpC2 acts as a transcriptional repressor and accounts for the significant upregulation of ClpC2, we have identified the transcriptional mechanism responsible for the regulation of *clpC2* gene expression in this and other studies.

The analysis of the Mtb ClpC2 structures that we present here suggests DNA interaction occurs via ClpC2 CTD and that the putative DNA-binding site is directly adjacent to or even partially overlapping the CymA-binding site, interfering with the binding of ClpC2 to the *clpC2* promoter and ultimately leading to derepression of the *clpC2* gene. The structural data suggest that CymA either prevents access of DNA to the DNA-binding site sterically or by triggering a conformational change in the ClpC2 C-terminal domains. Which signal might trigger the derepression of *clpC2* in other stress and adaptive responses requires further investigation. It is possible that secondary metabolites could act in place of CymA-binding, eliciting similar conformational changes in the C-terminal ClpC2 domains.

A similar distribution of positive charges as in the putative DNA-binding region of ClpC2 is observed in the corresponding region of ClpC1, prompting us to test ClpC1 in an EMSA experiment using the DNA probe containing the *clpC2* operator site. ClpC1 exhibited an affinity with a K_D_ in the low micromolar range, which constitutes at least two orders of magnitude lower affinity than exhibited by ClpC2 (Supplementary Fig. 11a). Furthermore, ClpC1 shows little sequence specificity for the *clpC2* operator site (Supplementary Fig. 11b, 11c). This is in accordance with previous reports of unspecific interaction with DNA for the ClpC1 functional homologue, *E. coli* ClpA, which was suggested to play a role in the degradation of certain replication initiator proteins^50,51^.

Taken together, we show ClpC2 presents an alternative binding site for CymA and that this, combined with the pronounced upregulation of ClpC2, helps to protect the cell from CymA-induced toxicity. Our work contributes to the growing understanding of the Clp complexes as drug targets and offers insight into the complexities of CymA-induced toxicity.

## Materials and methods

### Bacterial strains and growth conditions

*Mycobacterium smegmatis* MC^2^155 SMR5 and derived strains were routinely grown at 37 °C with shaking in DIFCO™ Middlebrook7H9 broth (BD) supplemented with 0.05% Tween 80 and 0.2% glycerol. The antibiotics apramycin (50 µg/ml) or hygromycin B (100 µg/ml) were added as needed. CymA (dissolved in DMSO) was added to the media as indicated and the DMSO concentration was matched in the relevant control experiments. The DMSO concentration of cultures was maintained at less than 1% in all instances.

### Protein expression and protein purification

All proteins were expressed in *E. coli* Rosetta (DE3) by isopropyl-β-D-thiogalactopyranosid (IPTG) induction in LB media at 20 °C overnight. Cells were harvested then resuspended in lysis buffer (50 mM HEPES-KOH pH 7.5, 300 mM KCl, 5 mM MgCl_2_, DNaseI, 1 mM PMSF, 1× Roche protease inhibitor cocktail) and lysed using a Microfluidizer M110-L (Microfluidics). Lysate was clarified by centrifugation in a SS34 rotor (ThermoScientific) at 20,000 rpm for 1 h at 4 °C. Untagged Mtb ClpC2 WT, ClpC2 R57A and ClpC2 R185A/R189A variants were precipitated by adding (NH_4_)_2_SO_4_ to 35% saturation followed by resolubilisation and dialysis into 50 mM HEPES-KOH pH 7.5, 75 mM KCl, 2 mM EDTA, 10% glycerol then subjected to anion exchange chromatography (Source Q column) using a salt gradient of 0.1–1 M KCl. Fractions containing ClpC2 were selected following SDS-PAGE and adjusted to 50 mM HEPES-KOH pH 7.5, 25% (NH_4_)_2_SO_4_, 150 KCl, 10% glycerol and further purified by hydrophobic interaction chromatography using a Butyl Sepharose 4 Fast Flow column with a 25–0% (NH_4_)_2_SO_4_ gradient. ClpC2 fractions were pooled and loaded onto a Superdex 75 16/600 column (GE Healthcare) for further purification in buffer GF (50 mM HEPES-KOH pH 7.5, 150 mM KCl, 10% glycerol). Far-UV CD spectra of the ClpC2 variants were recorded and compared to the spectrum of ClpC2 WT to ensure the variants are folded (Supplementary Fig. 12).

Lysate of untagged Mtb ClpC1 was diluted and pH-adjusted to 50 mM HEPES-KOH pH 7.1, 75 mM KCl, 2 mM EDTA, 10% glycerol and loaded onto a Fast Flow Q anion exchange column and purified using a salt gradient of 0.1–0.5 M KCl. Fractions containing ClpC1 were pooled and ClpC1 precipitated by adding (NH_4_)_2_SO_4_ to 40% saturation and then resolubilised and dialysed into 50 mM HEPES-KOH pH 7.1, 75 mM KCl, 2 mM EDTA, 10% glycerol. Soluble ClpC1 was again subjected to anion exchange chromatography (Source Q column) using a salt gradient of 0.1–0.5 M KCl followed by (NH_4_)_2_SO_4_ precipitation (40% saturation). ClpC1 protein was resuspended and dialysed into 50 mM HEPES-KOH pH 7.1, 150 mM KCl, 10% glycerol and loaded onto a HiLoad Superdex 200 26/600 column (GE Healthcare).

Recombinantly expressed ClpP1 and ClpP2 with C-terminal tetra-histidine tags (His_4_) were each purified by immobilised metal affinity chromatography (IMAC) using Ni^2+^- IMAC Sepharose 6 FF resin (GE Healthcare) followed by further purification on a Superose 6 column (GE Healthcare) in 50 mM HEPES-KOH pH 7.5, 300 mM NaCl, 10% glycerol. ClpP1P2 N-terminal propeptide processing was achieved by overnight incubation of equimolar concentrations of ClpP1-His_4_ and ClpP2-His_4_ at room temperature in 25 mM HEPES-KOH pH 7.5, 100 mM KCl, 5 mM MgCl_2_, 0.1 mM EDTA, 10% glycerol in the presence of 1 mM benzyloxycarbonyl-l-leucyl-l-leucinal (PeptaNova). Processed ClpP1P2 was further purified by gel filtration using a Superdex 200 column.

Both ClpC1 residues 1-145 (ClpC1 NTD) and ClpC2 residues 94-252 (ClpC2^94–252^) were expressed with an N-terminal deca-histidine tag (His_10_) followed by both the tobacco etch virus (TEV) and human rhinovirus 3c protease (3c) cleavage sites. His_10_-tev-3c-ClpC1 NTD and His_10_-tev-3c-ClpC2^94–252^ were first purified by IMAC using Ni^2+^- IMAC Sepharose 6 FF resin and both proteins cleaved overnight at 4 °C with TEV protease. ClpC1 NTD and ClpC2^94–252^ were further purified by Ni^2+^-NTA chromatography and the flow-through was collected and loaded onto a Superdex 75 16/600 column in buffer GF. All proteins were flash-frozen in liquid nitrogen and stored at −20 °C.

### Circular dichroism

Far-UV CD spectra of ClpC2 WT and ClpC2 variants R57A and R185A/R189A were recorded on a JASCO J-810 CD spectropolarimeter at a concentration of 8 µM. The spectra were measured at 23 °C in 50 mM Na_2_HPO_4_-NaH_2_PO_4_ (pH 7.4), 150 mM KCl in a 0.1 cm quartz cuvette. The recorded spectra were averages of 10 measurements and were corrected for buffer background.

### Isothermal titration calorimetry

ClpC1 NTD and ClpC2 were dialysed into ITC buffer (50 mM HEPES-KOH pH 7.5, 150 mM KCl, 9% DMSO, 1 mM EDTA) and their concentrations were determined by measuring absorbance at 280 nm. CymA was diluted into ITC buffer and care was taken to maintain the same concentration of DMSO as for the dialysed proteins. 50 μM (monomer concentration) of either ClpC1 NTD or ClpC2 was titrated into 5 μM CymA (1.4 ml) at 37 °C beginning with a 2 μl injection followed by 39 injections of 5 μl injections at intervals of 180 seconds using a VP-ITC instrument (MicroCal). Data were analysed using Origin v.7.0383 (OriginLab) with a one-site binding model.

### Degradation assay

Degradation of FITC-casein (Sigma) was carried out in 50 mM HEPES-KOH pH 7.1, 150 mM KCl, 20 mM MgCl_2_, 10% glycerol by 0.5 μM ClpC1 hexamer and 0.8 μM ClpP1P2 tetradecamer with the addition of 0.4 mM activator peptide benzyloxycarbonyl-L-leucyl-L-leucinal (PeptaNova), 0.5 mM DTT, 1 U/ml creatine phosphokinase, 20 mM phosphocreatine, 10 mM ATP at 37 °C. The CymA-stimulated degradation of FITC-casein (0.5 μM) was performed in the presence of 2 μM CymA and 0–18 μM ClpC2. Whereas the effect of an excess of ClpC2 on the degradation of FITC-casein (10 μM) was carried out in the presence of 100 μM ClpC2. Fluorescence intensity increases upon degradation of FITC-casein as a consequence of fluorophore dequenching was monitored using the BioTek Synergy 2 Plate Reader with a 485/20 nm band-pass emission filter and a 528/20 nm band-pass excitation filter. Rates of degradation were determined from the initial slopes and used to determine the CymA-induced stimulation of ClpC1P activity at the different concentrations of ClpC2.

### Protein crystallisation

Both ClpC2 (full length) and ClpC2^94–252^ were dialysed into 20 mM HEPES-KOH pH 7.5, 20 mM KCl before crystallisation by sitting drop vapour diffusion. Full-length ClpC2 was added to the crystallisation drop and took ~31 days to form crystals in 25% w/v PEG 3350, 0.1 M Tris-HCl pH 8.5. Crystals were soaked in cryo-protectant (35% w/v PEG 3340, 0.1 M Tris-HCl pH 8.5) prior to flash-freezing in liquid nitrogen. CymA was added 1.1-fold in molar excess to ClpC2^94–252^ and the complex concentrated to 12 mg/ml. The ClpC2^94–252^-CymA complex crystallised in 100 mM sodium acetate-HCl pH 4.5, 30% (w/v) PEG 8 K, 200 mM LiSO_4_. Crystals were allowed to grow for five days then soaked in cryo-protectant (100 mM sodium acetate-HCl pH 4.5, 30% (w/v) PEG 8 K, 200 mM LiSO_4_, 15% v/v glycerol) and flash frozen in liquid nitrogen.

### Data collection, structure determination, model building and refinement

X-ray diffraction data sets were collected at beamlines X06SA and X06DA of the Swiss Light Source (Paul Scherrer Institute, Villigen, Switzerland) and were indexed and integrated using XDS^52^. Data merging and scaling was carried out using the programme AIMLESS^53^ from the CCP4 suite^54^. The phase problem of ClpC2^4–72^ was solved using ARCIMBOLDO^37^ with default settings and search of nine copies of a helix containing 14 residues. The output poly-Ala model was then subject to auto-building using AutoBuild software in Phenix^55^ provided with the ClpC2 protein sequence. The output atomic model was further improved by iterative model building in COOT^56^ and refinement in Phenix.refine^57^. The crystal structure of ClpC2^94–252^-CymA was determined by molecular replacement with the programme Phaser^58^ using the previously solved structure of Mtb ClpC1 NTD (PDB 3WDC) as a search model excluding any ligand. The atomic model was first improved by AutoBuild and then by iterative model building in COOT and refinement in Phenix.refine. Statistics of both structures are summarised in Table 1. All figures were generated with PyMol (The PyMol Molecular Graphics System, version 2.4.1, Schrodinger, LLC) or UCSF Chimera (version 1.15).

### Generation of Msm *clpC2* knockout strain

A suicide plasmid for the markerless deletion of *clpC2* was constructed by amplifying 1500 bp upstream and 1500 bp downstream of *clpC2* ORF in Msm genomic DNA using primers listed in Supplementary Table 1a and assembling the PCR fragments into a XmnI digested pGOAL19 plasmid (Addgene plasmid #20190) using NEBuilder HiFi DNA Assembly (New England Biolabs). Following construction of the suicide plasmid, 200 μl Msm electrocompetent cells were transformed by electroporation with 1 μg UV-irradiated plasmid in a 0.2 cm cuvette (BioRad) and a pulse of 2.5 kV applied using a BioRad GenePulser. Electroporated cells were allowed to recover for 5 h at 37 °C in 7H9 and then plated on 7H10 agar plates with added hygromycin. Once colonies began to appear, the agar was underlayed with 0.4% X-Gal to allow for the identification of single-crossover colonies (SCOs). SCOs (blue colonies) were selected and grown in 7H9 at 37 °C before plating on 7H10 agar plates supplemented with 2% (w/v) sucrose followed by continued incubation at 37 °C then underlaying the agar with 0.4% X-Gal. Putative double-crossover colonies were identified by their white colour and screened by PCR for the complete deletion of *clpC2*.

### Growth curves

Msm cultures were diluted to an OD_600_ of 0.005 in 7H9. Each culture was split between two flasks: CymA (150 nM) was added to one flask and the equivalent volume of DMSO (0.03%) was added to the other. The cultures were grown at 37 °C with shaking (200 rpm) and the culture growth monitored by periodically measuring the OD_600_.

### Western blot analysis

Primary antibodies used for Western blotting are the polyclonal α-ClpC1 (1:10000 dilution) and α-ClpC2 (1:10000 dilution) antibodies raised in rabbits against Mtb ClpC1 and ClpC2, respectively (Eurogentec) and the commercially available monoclonal α-RpoB antibody (BioLegend, 1:10000 dilution, clone 8RB13) raised in mouse. Secondary antibodies were horseradish peroxidase (HRP)-conjugated α-rabbit (abcam, 1:10000 dilution, ab6721) and α-mouse (abcam, 1:10000 dilution, ab6789) antibody. CymA (150 nM) was added to a Msm culture (OD_600_ of ~0.5) grown in 7H9 media. Cells were harvested by centrifugation at the indicated time points and resuspended in 800 µl 50 mM HEPES-KOH, 150 mM KCl, 1 mM PMSF, 1× cOmplete, EDTA-free, protease inhibitor cocktail (Roche). Resuspended cells were lysed in 2 ml screw cap tubes containing 500–700 mg 0.15 mm zirconium oxide beads (NextAdvance) using a Minilys personal homogeniser (Bertin Technologies). The lysate was clarified by centrifugation for 10 minutes at 20,000 × *g* and the total protein concentration measured using a NanoDrop™ One spectrophotometer (ThermoScientific). 50 μg protein was separated on a 15% polyacrylamide gel and transferred to a PVDF Immobilon-P membrane (Merck) using a BioRad Trans-Blot SD semidry transfer cell at 20 V for 60 minutes. The PVDF membrane was first blocked for 1 hour with 1% PVP-40 in PBS-T (20 mM Na_2_HPO_4_, 2 mM KH_2_PO_4_, 137 mM NaCl, 2.7 mM KCl, 0.05% Tween 20) then incubated with the primary antibody in PBS-T with 1% PVP-40 before washing three times in PBS-T and applying the secondary antibody in PBS-T with 1% PVP-40 for 1 hour. The membrane was washed four times for 5 minutes each in PBS-T and the HRP-conjugated secondary antibodies detected using the ECL substrate (BioRad Clarity Western Substrate). Blots were imaged on an Amersham Imager 600 (GE Healthcare).

### Quantitative Western blot analysis

Msm cultures were grown to OD_600_ of 0.5 and 150 nM CymA (or equivalent volume of DMSO) added to the cultures. At the indicated time points 20 OD equivalents of cell culture were pelleted and the cells were lysed following the above protocol. The total protein concentration in the cleared lysates was determined by Bradford Assay and their volume adjusted to a nominal concentration of 2 mg/ml. From here onwards the procedure is the same as stated in the protocol for Western blotting above. Protein levels present at the indicated time points were determined densitometrically from the blots using GelAnalyzer 19.1 by comparison to a standard curve prepared from purified ClpC1 and ClpC2. The ClpC1 and ClpC2 protein levels are reported relative to ClpC1 levels present at the 0 h timepoint.

### RT-qPCR

Msm cultures were grown in 7H9 at 37 °C to the stated OD_600_ before application of the stressor followed by continued incubation for the indicated time. Cells were harvested by centrifugation and then lysed in 2 ml screw cap tubes containing 500–700 mg 0.15 mm zirconium oxide beads using a Minilys personal homogeniser. The lysate was cleared by centrifugation at 20,000 × *g* for 10 minutes and the total RNA purified using a SPLIT RNA Extraction kit (Lexogen) and following the manufacturer’s instructions. Removal of DNA was achieved using the Turbo DNase kit (ThermoFisher Scientific) and the RNA concentration measured using the NanoDrop™ One spectrophotometer. 100 ng RNA was reverse transcribed to complementary DNA (cDNA) using iScript Select cDNA Synthesis kit (BioRad). Primers for qPCR were designed using Primer3 software^59^ and are listed in Supplementary Table 1b. qPCR was performed using the Kapa Sybr Fast qPCR kit (Roche) in a BioRad CFX96 thermocycler with an initial 5 minutes at 95 °C step followed by 40 cycles of 95 °C for 10 seconds, 60 °C for 10 seconds, 72 °C for 12 seconds. Data were analysed using BioRad CFX Maestro Software. Relative gene expression was determined using the 2^-^^ΔΔCt^ method and with *rpoB* or *sigA* as the reference gene.

### DNA pull-down assay

Msm was grown in 7H9 to an OD_600_ of 0.5 before addition of 1 µM CymA (or DMSO for the control Msm culture) and incubated for a further 2 h. Cells were pelleted by centrifugation, resuspended in BS/THES (44.3% THES, 20% BS) with added cOmplete protease inhibitor cocktail (Roche) and lysed by bead beating with a Minilys personal homogeniser and 500–700 mg 0.15 mm zirconium oxide beads in 2 ml screw cap tubes. Lysate was sonicated with a Misonix Sonicator 3000 with three cycles of 15 seconds sonication followed by 1 minute incubation on ice and the lysate clarified by centrifugation at 20,000 × *g* for 20 minutes. The clarified lysate was diluted to 90 ODs/ml and incubated with streptavidin-conjugated agarose beads for 1 hour at room temperature and the precleared lysate separated from the agarose beads by centrifugation at 700 × *g* for 1 minute. The DNA probe was prepared by amplifying 300 bp upstream *clpC2* (or intragenic DNA sequence for the control probe) using biotinylated primers (Supplementary Table 1c). 40 µg DNA was immobilised on 1 mg Dynabeads MyOne Streptavidin C1 (Invitrogen) using a magnetic stand and washed twice with B/W buffer (10 mM Tris-HCl pH 7.5, 1 mM EDTA, 2 M NaCl) followed by three washes with TE buffer (0.5 M Tris-HCl pH 8, 1 mM EDTA) and two washes with BS/THES. The DNA-bound beads were incubated for 30 minutes with 10 µg/ml salmon sperm DNA in BS/THES. DNA-bound beads were resuspended in 750 µl fresh BS/THES containing 50 µg/ml salmon sperm DNA to which 200 µl precleared lysate was added and the mixture incubated for 90 minutes at room temperature with agitation. The beads were washed five times with 500 µl BS/THES followed by a wash with 25 mM Tris-HCl pH 7.5, 100 mM NaCl and then a wash with 25 mM Tris-HCl pH 7.5, 200 mM NaCl. Proteins bound to the immobilised DNA were recovered at the Functional Genomics Center Zurich by incubation with 500 ng trypsin in digestion buffer (10 mM Tris pH 8.2, 2 mM CaCl_2_) at 60 °C for 30 minutes. The supernatant was collected following trypsin digest and combined with the supernatant produced from incubation of the beads with 0.1% TFA/ 50% acetonitrile then dried and dissolved in 20 μl ddH_2_O + 0.1% formic acid in preparation for liquid chromatography-tandem mass spectrometry (LC-MS/MS). LC-MS/MS was carried out using a nanoAcquity UPLC coupled to a Q-Exactive mass spectrometer (Thermo) and data analysed using the Mascot search engine (Matrixscience) where spectra were searched against *Mycobacterium smegmatis* (Swissprot) for the identification of proteins. Relative amounts of ClpC2 co-purified and detected with either the control DNA or *clpC2* promoter DNA sequence were determined from spectral counts.

### Electrophoretic mobility shift assay

DNA probes were synthesised by Microsynth AG and are listed in Supplementary Table 1d. To prepare double-stranded DNA, complementary strands were annealed by heating equimolar concentrations to 95 °C in a thermocycler and slowly cooled to 25 °C by reducing the temperature by 1 °C every minute. DNA-binding reactions were prepared by mixing the DNA probe with the indicated concentrations of protein in 5 mM HEPES-KOH pH 7.5, 30 mM KCl, 1% Glycerol (v/v), 10 mM MgCl_2_ and incubated at room temperature for 20 minutes before loading onto a 12% polyacrylamide gel. To analyse the effects of CymA on DNA-binding, 1 µM CymA was added to the binding reaction and the DMSO concentration matched for the control without CymA and the gel band density was quantified using GelAnalyzer 19.1 software.

### Statistics and reproducibility

Statistical analysis and graphs were prepared using GraphPad Prism 9.3.1 software. Data are presented as mean ± standard deviation (SD) where relevant and the number of replicates is described in the respective figure legend. *P* values <0.05 were considered significant and statistical tests used are stated in the figure legends.

### Reporting summary

Further information on research design is available in the Nature Portfolio Reporting Summary linked to this article.

## Supplementary information

Supplementary Information

Description of Additional Supplementary Files

Supplementary Data 1

Supplementary Data 2

Reporting Summary

## Data Availability

The atomic coordinates and structure factors have been deposited in the Protein Data Bank database under the accession codes 8ADA (ClpC2^4–72^) and 8AD9 (ClpC2^94–252^-CymA). All other data are either contained in the paper or in the associated supplementary information files. Source data underlying graphs and uncropped images of gels and blots presented in this paper are provided in Supplementary Data 2.
