# Supplementary material for: ClpC2 protects mycobacteria against a natural antibiotic targeting ClpC1-dependent protein degradation
Source: Commun Biol. 2023 Mar 21;6:301. doi: 10.1038/s42003-023-04658-9 (PMC10030653; doi:10.1038/s42003-023-04658-9)
Supplement: Supplementary file 1 — Supplementary Information [file 42003_2023_4658_MOESM1_ESM.pdf]

Supplementary Information

ClpC2 protects mycobacteria against a natural antibiotic targeting ClpC1-dependent protein degradation

Gabrielle Taylor[a], Hengjun Cui[a], Julia Leodolter[a]*, Christoph Giese[a], Eilika Weber-Ban[a]#

[a]ETH Zurich, Institute of Molecular Biology & Biophysics, CH-8093 Zurich, Switzerland

#Address correspondence to Eilika Weber-Ban, eilika@mol.biol.ethz.ch.
*Present address: Research Institute of Molecular Pathology (IMP), Vienna, Austria.

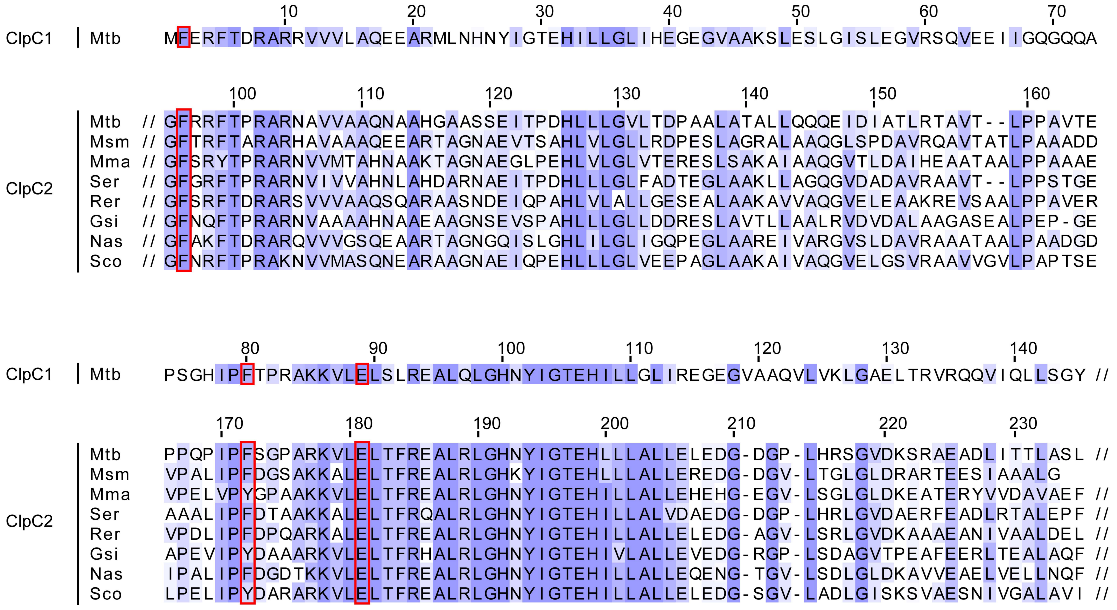

Supplementary Figure 1. Alignment of Mtb ClpC1 NTD and ClpC2 C-terminal regions from a selection of actinobacteria. Residues are coloured according to percentage identity and those residues associated with binding of CymA in ClpC1 (Phe2, Phe80 and Glu89) are bordered in red. Mtb, *Mycobacterium tuberculosis*; Msm, *Mycobacterium smegmatis*; Mma, *Modestobacter marinus*; Ser, *Saccharopolyspora erythraea*; Rer, *Rhodococcus erythropolis*; Gsi, *Gordonia sihwensis*; Nas, *Nocardia asteroides*; Sco, *Streptomyces coelicolor*.

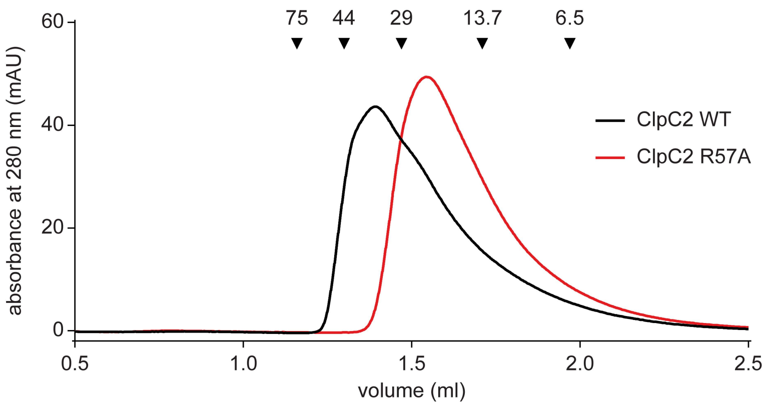

Supplementary Figure 2. Elution profiles of ClpC2 WT and ClpC2 R57A variant as analysed by size-exclusion chromatography on a Superdex 75 Increase 5/150 GL column.

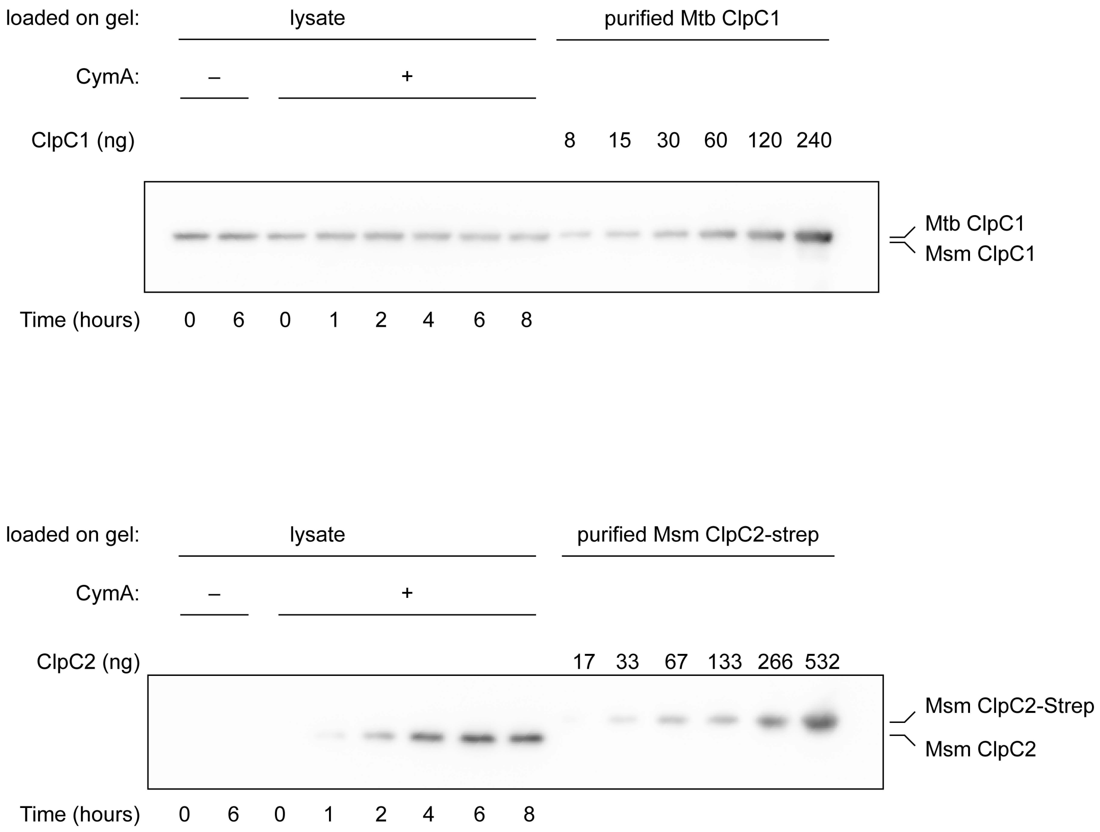

Supplementary Figure 3. Representative immunoblots used in quantifying ClpC1 and ClpC2 protein levels for Fig. 3d. Msm lysate samples were prepared at various timepoints following addition of 150 nM CymA. Msm ClpC1 and Msm ClpC2 were detected with α-ClpC1 (top panel) and α-ClpC2 (bottom panel) antibodies and protein levels quantified in reference to a standard curve using either purified Mtb ClpC1 or Msm ClpC2-strep, respectively.

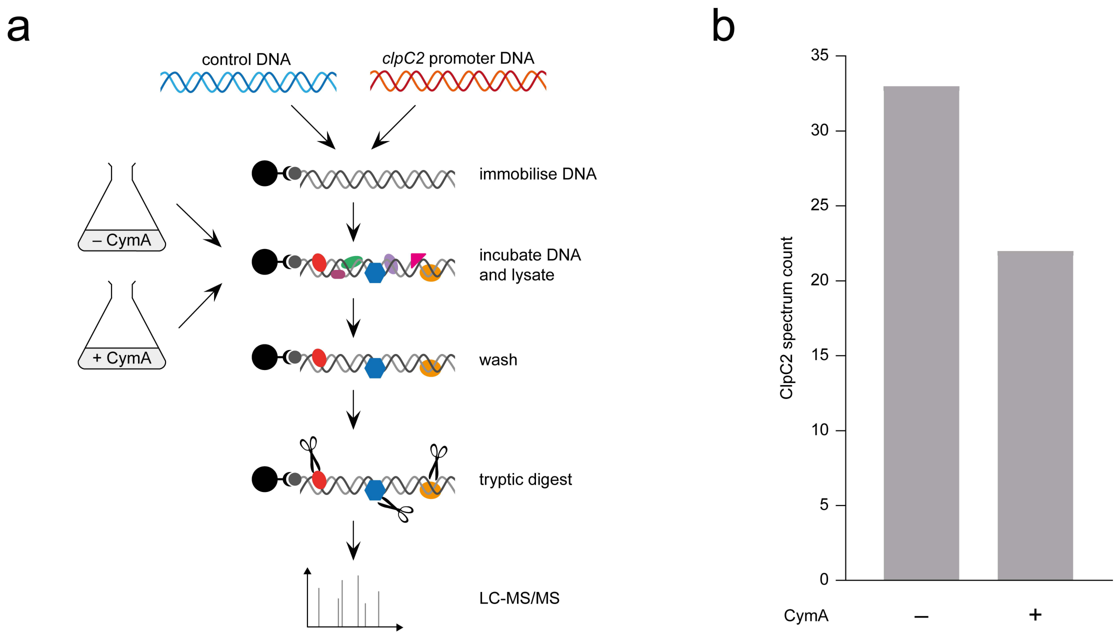

Supplementary Figure 4. Identification of transcription factors controlling *clpC2* expression in response to CymA. a Overview of the DNA pulldown assay used to identify transcription factors regulating *clpC2* expression. Biotin-labelled DNA containing the *clpC2* promoter sequence or intragenic DNA (control DNA) was immobilised on streptavidin beads. The immobilised DNA was incubated with Msm lysate that was grown in either the absence or presence of CymA before washing the beads with low salt buffers to help remove non-specific interactors. Proteins bound to the DNA were subjected to tryptic digestion and identified by LC-MS/MS. **b** Quantification of ClpC2 peptides detected by LC-MS/MS in a DNA pull-down assay using immobilised *clpC2* promoter DNA as bait.

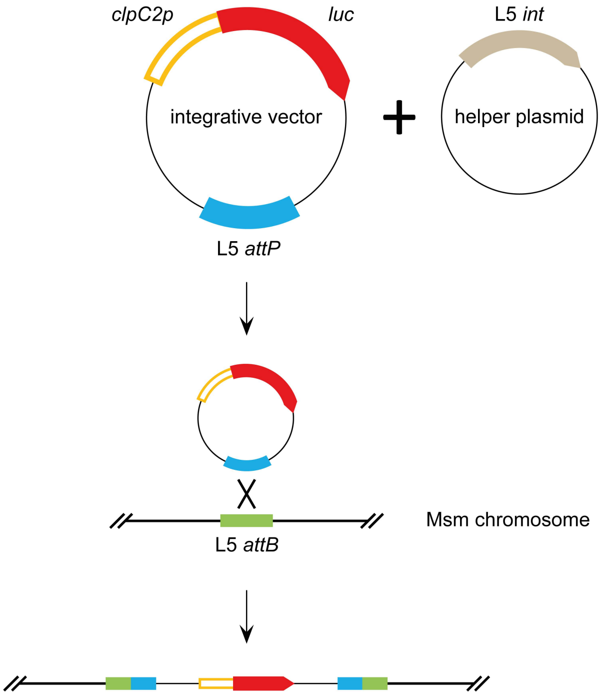

Supplementary Figure 5. Schematic diagram representing the generation of strains used to analyse the effect of ClpC2 on its own expression. The luciferase gene (*luc*) under the control of the *clpC2* promoter (*clpC2p*) was introduced into the genomes of both WT and Δ*clpC2* strains of Msm via an integrative vector. Stable integration of the integrative vector was achieved using a non-replicative helper vector encoding the L5 integrase (*int*) allowing for recombination between the L5 *attP* site on the integrative vector and the L5 *attB* site in the Msm genome.

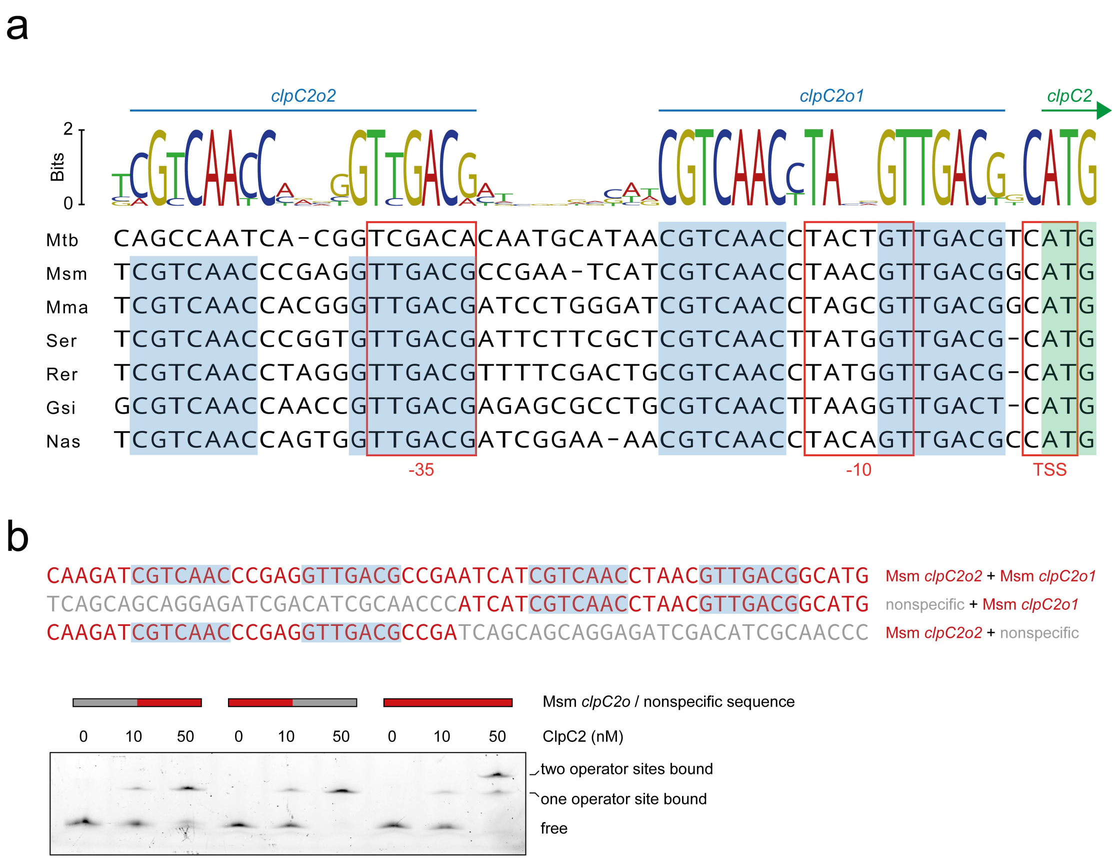

Supplementary Figure 6. *clpC2* promoter region. a Promoter sequences from diverse actinobacterial species. Indicated with a red border are the -10 and -35 regions as well as the transcription start site (TSS). The conserved putative *clpC2* operator sites (*clpC2o1* and *clpC2o2*) and *clpC2* start codon are highlighted in blue and green, respectively. Mtb, *Mycobacterium tuberculosis*; Msm, *Mycobacterium smegmatis*; Mma, *Modestobacter marinus*; Ser, *Saccharopolyspora erythraea*; Rer, *Rhodococcus erythropolis*; Gsi, *Gordonia sihwensis*; Nas, *Nocardia asteroides*. **b** An EMSA using DNA sequences containing the Msm *clpC2* promoter region with either both *clpC2* operator sites present or with one *clpC2o* replaced with a nonspecific sequence. All DNA sequences are of equal length (58 bp) and were incubated with the indicated concentrations of ClpC2. DNA sequences are presented above with Msm *clpC2o* sequence coloured red and the nonspecific sequence coloured grey.

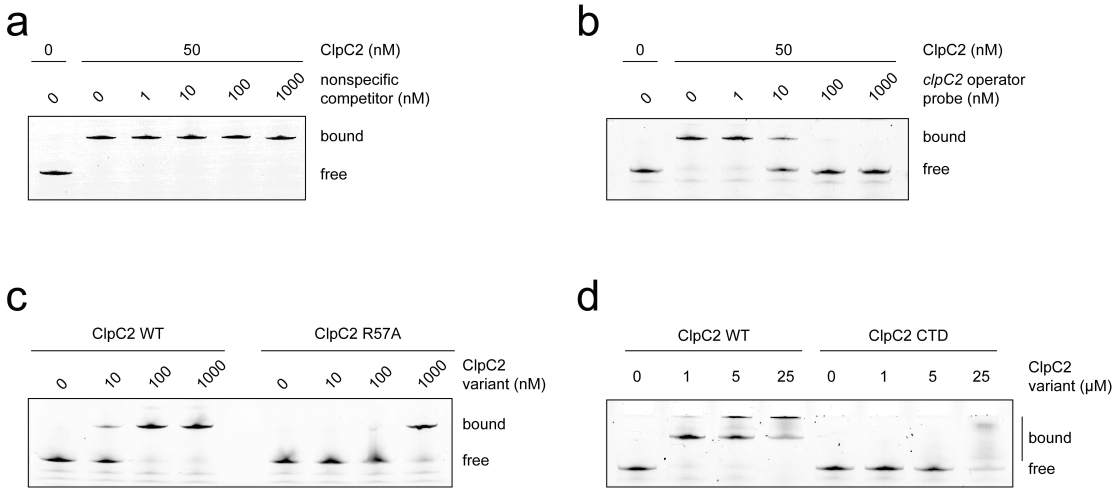

Supplementary Figure 7. Characterization of ClpC2-DNA interaction and sequence specificity. a 1-1000 nM unlabelled nonspecific competitor DNA (23 bp) was incubated with 50 nM ClpC2 prior to the addition of 1 nM labelled DNA probe encompassing the *clpC2* operator site. **b** ClpC2 was incubated with 1 nM labelled DNA probe (23 bp) containing the *clpC2* operator sequence and 1-1000 nM unlabelled DNA probe also containing the *clpC2* operator sequence. **c** 10-1000 nM of both ClpC2 WT and ClpC2 R57A were each incubated with 5 nM labelled DNA probe. **d** 5 nM labelled DNA probe was incubated with either ClpC2 WT or ClpC2 CTD at the indicated concentrations.

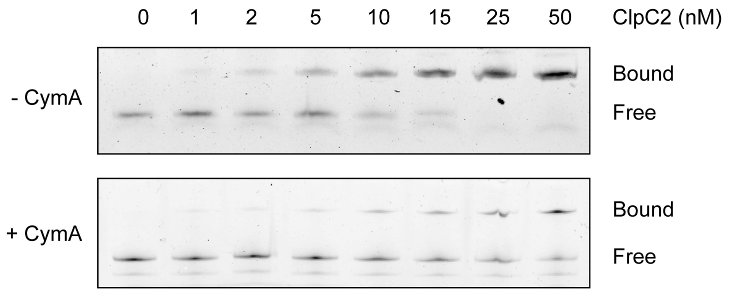

Supplementary Figure 8. Representative gels used in preparation of figure 4d showing the influence of CymA on ClpC2 DNA binding activity.

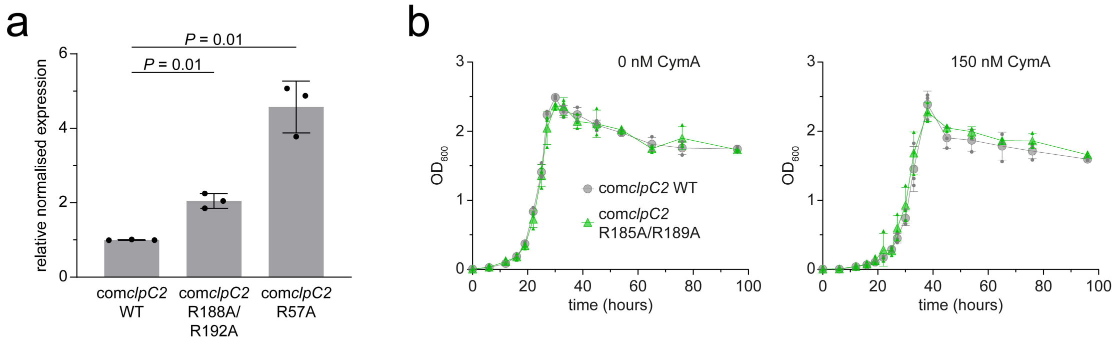

Supplementary Figure 9. The effect of ClpC2 variants on *clpC2* expression levels and Msm growth behaviour. a Quantification of *clpC2* mRNA levels of Δ*clpC2* complemented with either wild-type *clpC2* (com*clpC2* WT), *clpC2* R185A/R189A (com*clpC2* R188A/R192A) or *clpC2* R57A (com*clpC2* R57A). Strains were grown under standard growth conditions to an OD_{600} of 0.8 and RNA extracted for RT-qPCR analysis. *rpoB* served as the reference gene. Presented are the mean values ± S.D. of biological triplicates and statistical analysis by unpaired two-tailed Student's *t*-test. **b** Growth curves of Δ*clpC2* complemented with either wild-type *clpC2* (com*clpC2* WT, grey circles) or *clpC2* R185A/R189A (com*clpC2* R185A/R189A, green triangles) grown in either the absence or presence of 150 nM CymA.

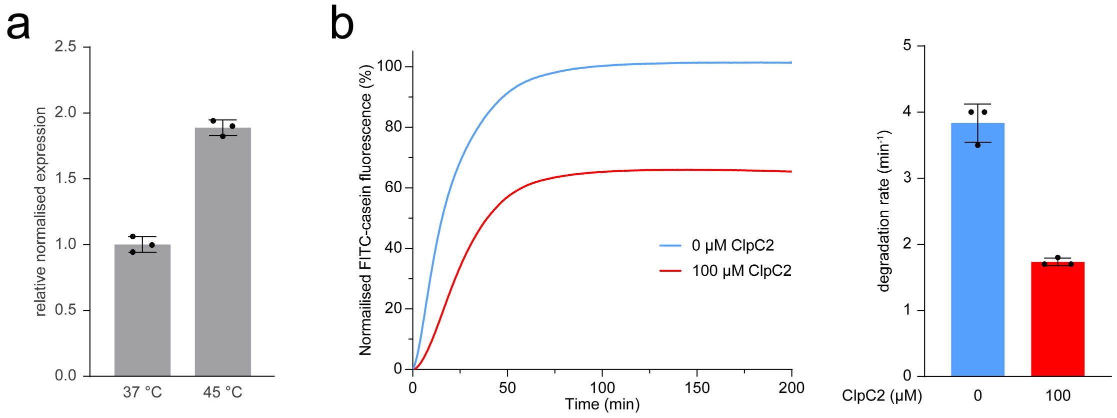

Supplementary Figure 10. Expression of *clpC2* is induced during heat shock. a RT-qPCR analysis of *clpC2* mRNA levels under heat stress conditions. Msm was grown to an OD_{600} of 1.5 followed by incubation at either 37 °C or 45 °C for 30 minutes and total RNA extraction. *clpC2* mRNA levels were analysed using the housekeeping gene *sigA* as reference. Statistical analysis performed using the two-tailed unpaired Student's *t*-test; *P* value < 0.0001. **b** FITC-casein degradation mediated by ClpC1P in either the absence or presence of an excess of ClpC2. Time course of FITC-casein degradation by 0.5 µM ClpC1 hexamer/0.8 µM ClpP1P2 tetradecamer in either the absence (blue) or presence (red) of 100 µM ClpC2 (left) and the determined initial rates (right).

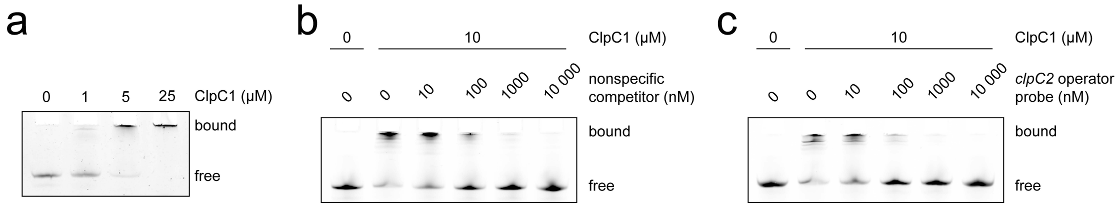

Supplementary Figure 11. ClpC1 binds unspecifically to DNA. a EMSA analysis of ClpC1 binding to DNA. 1-25 µM ClpC1 was incubated with 0.5 nM DNA probe. **b** and **c** ClpC1 was incubated with either 41 bp unlabelled nonspecific competitor DNA (**b**) or 41 bp unlabelled DNA probe including the *clpC2* operator site (**c**) at the specified concentrations before addition of 10 nM labelled DNA probe that includes the *clpC2* operator site.

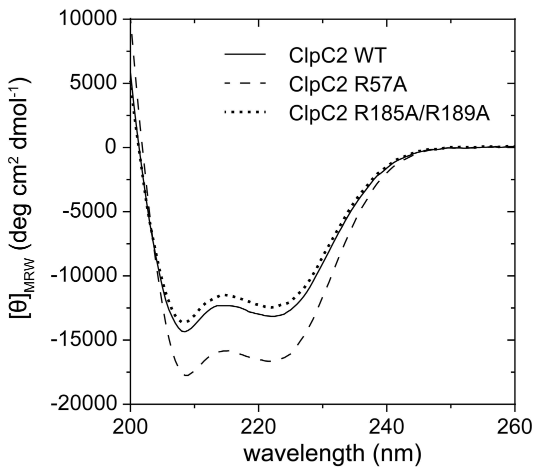

Supplementary Figure 12. The CD spectra of recombinantly expressed ClpC2 WT, ClpC2 R57A and ClpC2 R185/R189A show signatures of natively folded proteins.

Supplementary Table 1. Selected oligonucleotides used in this study.

a Primers used to generate the suicide plasmid for the preparation of Msm Δ*clpC2* strain. Overhangs for NEBuilder are in lower case.

Name	Sequence (5' - 3')	Purpose
clpC2_upstream_Fw	cgccccgaagaacgGTCAGAACGAG GCGTCGT	Amplifies region upstream of Msm *clpC2*
clpC2_upstream_Rv	cgcgtttcactccgcTTCGACCATGCC GTCAAC	
clpC2_downstream_Fw	GCGGAGTGAAACGCGGGA	Amplifies region downstream of Msm *clpC2*
clpC2_downsteam_Rv	gtgctcatcattggaaaaGAAGCCGTG CTTGGAGTAG	

b Primers used in RT-qPCR

Name	Sequence (5' - 3')	Purpose
clpC2_qPCR_Fw	ATCGCCGACCACCTGATC	Detects Msm *clpC2* transcripts
clpC2_qPCR_Rv	CTTGGGGACGAAACGCTTC	
rpoB_qPCR_Fw	CGTCACGGCTGAGTTCATC	Detects Msm *rpoB* transcripts
rpoB_qPCR_Rv	CCTTTTCGGTCATCATCGGG	
sigA_qPCR_Fw	GACTACACCAAGGGCTACAAG	Detects Msm *sigA* transcripts
sigA_qPCR_Rv	TTGATCACCTCGACCATGTG	
luc_qPCR_Fw	CGCCGGGCTTTAATGAGTAT	Detects firefly *luc* transcripts
luc_qPCR_Rv	CCCGACGAGTTCATGATCAA	

c Primers used to generate biotin-labelled DNA probes for use in the DNA pull-down assay.

Name	Sequence (5' - 3')	Modification	Purpose
clpC2_DNA_PD_Fw	GTCCAACTGGGCGTACT G	5'-biotin	Amplifies *clpC2* promoter DNA in Msm
clpC2_DNA_PD_Rv	CATGCCGTCAACGTTAG GTTG		
control_DNA_PD_Fw	CGGATCGGAACCGGGC GTGGC	5'-biotin	Amplifies *tatC* intragenic DNA in Msm for use as control DNA probe
control_DNA_PD_Rv	CGCCGCTTCGCGATGGC GTTCG		

d Oligonucleotides used in electrophoretic mobility shift assay

Name	Sequence (5’ - 3’)	Modification	Purpose
clpC2_FAM_EMSA	ACAATGCATAACGTCAACCTACTGTTGACGTCATGCCGGAG	5’-FAM	41 bp DNA incorporating the *clpC2* operator site
nonspecific_EMSA_23bp	GATCACTTAATCGGCCACTTCGT		23 bp DNA for use as a nonspecific competitor in EMSA experiments involving ClpC2
specific_EMSA_23bp	AACGTCAACCTACTGTTGACGTC		23 bp DNA incorporating the *clpC2* operator site for use as a specific competitor in EMSA experiments involving ClpC2
nonspecific_EMSA_41bp	GTTGCTTCAGCAGCAGGAGATCGACATCGCAACCCTGCGTA		41 bp DNA for use as a nonspecific competitor in EMSA experiments involving ClpC1
specific_EMSA_41bp	ACAATGCATAACGTCAACCTACTGTTGACGTCATGCCGGAG		41 bp DNA incorporating the *clpC2* operator site for use as a specific competitor in EMSA experiments involving ClpC1
