# Supplementary material for: ClpC2 protects mycobacteria against a natural antibiotic targeting ClpC1-dependent protein degradation
Source: Commun Biol. 2023 Mar 21;6:301. doi: 10.1038/s42003-023-04658-9 (PMC10030653; doi:10.1038/s42003-023-04658-9)
Supplement: Supplementary file 2 — Description of Additional Supplementary Files [file 42003_2023_4658_MOESM2_ESM.pdf]

Description of Additional Supplementary Files

File name: Supplementary Data 1

Description: Proteins co-purified with either biotinylated clpC2 promoter or control intragenic DNA in the DNA-pulldown assay and detected by LC-MS/MS.

File name: Supplementary Data 2

Description: The source data behind graphs, electrophoretic mobility shift gels and Western blots presented in the paper.
