# Supplementary material for: ClpC2 protects mycobacteria against a natural antibiotic targeting ClpC1-dependent protein degradation
Source: Commun Biol. 2023 Mar 21;6:301. doi: 10.1038/s42003-023-04658-9 (PMC10030653; doi:10.1038/s42003-023-04658-9)
Supplement: Supplementary file 5 — Reporting Summary [file 42003_2023_4658_MOESM5_ESM.pdf]

nature portfolio

Corresponding author(s): Eilika Weber-Ban

Last updated by author(s): Jan 25, 2023

Reporting Summary

Nature Portfolio wishes to improve the reproducibility of the work that we publish. This form provides structure for consistency and transparency in reporting. For further information on Nature Portfolio policies, see our Editorial Policies and the Editorial Policy Checklist.

Statistics

For all statistical analyses, confirm that the following items are present in the figure legend, table legend, main text, or Methods section.

n/a	Confirmed	
☐	☒	The exact sample size (*n*) for each experimental group/condition, given as a discrete number and unit of measurement
☐	☒	A statement on whether measurements were taken from distinct samples or whether the same sample was measured repeatedly
☐	☒	The statistical test(s) used AND whether they are one- or two-sided<br>*Only common tests should be described solely by name; describe more complex techniques in the Methods section.*
☒	☐	A description of all covariates tested
☒	☐	A description of any assumptions or corrections, such as tests of normality and adjustment for multiple comparisons
☐	☒	A full description of the statistical parameters including central tendency (e.g. means) or other basic estimates (e.g. regression coefficient) AND variation (e.g. standard deviation) or associated estimates of uncertainty (e.g. confidence intervals)
☐	☒	For null hypothesis testing, the test statistic (e.g. *F*, *t*, *r*) with confidence intervals, effect sizes, degrees of freedom and *P* value noted<br>*Give P values as exact values whenever suitable.*
☒	☐	For Bayesian analysis, information on the choice of priors and Markov chain Monte Carlo settings
☒	☐	For hierarchical and complex designs, identification of the appropriate level for tests and full reporting of outcomes
☒	☐	Estimates of effect sizes (e.g. Cohen's *d*, Pearson's *r*), indicating how they were calculated

Our web collection on statistics for biologists contains articles on many of the points above.

Software and code

Policy information about availability of computer code

Data collection	Swiss Light Source (SLS, Paul-Scherrer-Institut, Villigen, Switzerland) in-house software for beamline operation
Data analysis	XDS (BUILT=20200417), AIMLESS 0.7.4, Phenix 1.19_4092, Phaser 2.8.3, Coot 0.9.4, PyMOL 2.4.1, UCSF Chimera 1.15, GelAnalyzer 19.1, GraphPad Prism 9.3.1

For manuscripts utilizing custom algorithms or software that are central to the research but not yet described in published literature, software must be made available to editors and reviewers. We strongly encourage code deposition in a community repository (e.g. GitHub). See the Nature Portfolio guidelines for submitting code & software for further information.

Data

Policy information about availability of data

All manuscripts must include a data availability statement. This statement should provide the following information, where applicable:

- Accession codes, unique identifiers, or web links for publicly available datasets
- A description of any restrictions on data availability
- For clinical datasets or third party data, please ensure that the statement adheres to our policy

Structural data are deposited in the protein data bank (pdb). All other datasets are contained in the main text or the supplement.

Human research participants

Policy information about studies involving human research participants and Sex and Gender in Research.

Reporting on sex and gender	N/A
Population characteristics	N/A
Recruitment	N/A
Ethics oversight	N/A

Note that full information on the approval of the study protocol must also be provided in the manuscript.

Field-specific reporting

Please select the one below that is the best fit for your research. If you are not sure, read the appropriate sections before making your selection.

☒ Life sciences ☐ Behavioural & social sciences ☐ Ecological, evolutionary & environmental sciences

For a reference copy of the document with all sections, see nature.com/documents/nr-reporting-summary-flat.pdf

Life sciences study design

All studies must disclose on these points even when the disclosure is negative.

Sample size	In vitro biochemical experiments were repeated individually three or more times. In vivo assays were repeated with three biological replicates.
Data exclusions	Data exclusion for X-ray analysis was based on CC1/2 > 0.6
Replication	Experiments were each repeated three or more times. Results were reproducible.
Randomization	Experiments were repeated individually.
Blinding	N/A

Reporting for specific materials, systems and methods

We require information from authors about some types of materials, experimental systems and methods used in many studies. Here, indicate whether each material, system or method listed is relevant to your study. If you are not sure if a list item applies to your research, read the appropriate section before selecting a response.

Materials & experimental systems

n/a	Involved in the study	
☐	☒	Antibodies
☒	☐	Eukaryotic cell lines
☒	☐	Palaeontology and archaeology
☒	☐	Animals and other organisms
☒	☐	Clinical data
☒	☐	Dual use research of concern

Methods

n/a	Involved in the study	
☒	☐	ChIP-seq
☒	☐	Flow cytometry
☒	☐	MRI-based neuroimaging

Antibodies

Antibodies used	α-RpoB (E. coli) monoclonal (mouse), BioLegend clone #8RB13 Cat. #663903<br>α-ClpC1 (M. tuberculosis) polyclonal (rabbit) was custom produced and purified<br>α-ClpC2 (M. tuberculosis) polyclonal (rabbit) was custom produced and purified
Validation	α-RpoB (E. coli) was validated by the manufactures via western blot analysis as outlined on its website.<br>α-Clp1 (M. tuberculosis) and α-Clp2 (M. tuberculosis) were validated by us via western blot analysis.
